# Detecting progressing events in multiple sclerosis clinics in real-time is possible: No

**DOI:** 10.1177/13524585251409547

**Published:** 2026-01-17

**Authors:** Isabel Voigt, Tjalf Ziemssen

**Affiliations:** Center of Clinical Neuroscience, Department of Neurology, Faculty of Medicine and University Hospital Carl Gustav Carus, TUD Dresden University of Technology, Dresden, Germany; Center of Clinical Neuroscience, Department of Neurology, Faculty of Medicine and University Hospital Carl Gustav Carus, TUD Dresden University of Technology, Dresden, Germany

There is growing agreement in the multiple sclerosis (MS) community on the importance of early detection of disease progression for timely intervention. Monitoring progressive events in real-time at MS clinics is appealing, but we argue it lacks clinical evidence. Despite advances in neuroimaging and digital tools, MS progression is inherently slow, multidimensional, and hard to assess at a single moment.^1–3^ Real-time detection—defined as the immediate identification of true irreversible worsening during a clinical visit—is still an aspirational goal, not a current reality. Although serial assessments can observe progression and trends may emerge with close monitoring, they do not constitute real-time detection.^4^ We contend that the biological complexities of progression, the limitations of existing clinical tools, the limitations of digital biomarkers, and the constraints of healthcare systems prevent reliable real-time detection at this time.

## Biological nature of MS progression: a slow-moving target

MS progresses with gradual accumulation of irreversible disability, independent of relapses, often occurring subtly over months or years and remaining unnoticed by patients and clinicians. Unlike acute relapses, progressive changes are not easily identified at a specific moment.^5^

Slow progression results from smoldering inflammation, chronic demyelination, axonal degeneration, and synaptic loss, driven by mechanisms that do not lead to dramatic clinical events. Instead, small, incremental changes accumulate, often obscured by neuroplasticity and daily variability. Factors like age-related comorbidities, fatigue, and mood can further complicate detection.^3,6,7^

These biological characteristics hinder real-time detection of MS progression, which is typically inferred retrospectively through sustained worsening observed over multiple visits. Even in detailed cohort studies like Multiple SclerosisBase (MSBase) and Comprehensive Longitudinal Investigation of Multiple Sclerosis at Brigham and Women’s Hospital (CLIMB), confirming progression requires several data points over months.^4^ Claiming that such events can be identified in a single clinical visit misrepresents the disease’s biology and the limitations of clinical assessment.

## Clinical tools lack real-time sensitivity

Routine clinical assessments for MS are not geared to detect progression immediately. The Expanded Disability Status Scale (EDSS), the primary outcome measure, has several limitations. It focuses on ambulation, lacks sensitivity to upper limb and cognitive changes, and suffers from inter-rater variability. EDSS was not designed for single-time-point assessments; even longitudinally, confirmation of progression requires 3 to 6months.^4^

Performance-based tests like the Timed 25-Foot Walk (T25FW), Nine-Hole Peg Test (9HPT), and Symbol Digit Modalities Test (SDMT) provide useful data, but have significant intraindividual variability. They require trend analysis to distinguish true progression from daily fluctuations and have limited sensitivity to meaningful change, especially in mildly disabled patients or cognitive domains.^5^

Clinicians may notice a deterioration in function during a visit, but without contextual data or previous measures, it’s challenging to determine whether this indicates true progression or temporary fluctuation.^5^ Relying on single-visit data for real-time progression assessments risks over- or under-diagnosis, leading to suboptimal patient care.

## Digital health tools: high frequency ≠ real-time diagnosis

The rapid rise of digital health technologies offers the potential for continuous and detailed MS assessment.^4,5^ While these tools could theoretically enable earlier detection of decline, they are not yet ready for clinical use.

Current digital biomarkers are largely unvalidated, suffer from data quality issues, and lack standardized thresholds for meaningful clinical change.^4^ Algorithms for detecting subtle changes in mobility or activity require contextualization, longitudinal data, and machine learning models still in development.^8^ Most tools require data collection over days or weeks, contradicting the notion of real-time detection during clinical visits.

Integrating promising indicators into clinical workflows poses challenges. Data acquisition and interpretation demand time, infrastructure, and trained personnel, which many clinics lack. Patient adherence varies, and false positives are a concern. Digital solutions do not enable definitive progression diagnosis during standard appointments. These tools may assist in long-term monitoring and suggest trends for further evaluation, but cannot provide immediate diagnostic certainty.^9^

## Real-world clinical constraints and systemic barriers

A barrier to real-time MS progression detection is the delivery of care. MS clinic visits are limited by time and resources, with patients seen every 6 to12 months and assessments occasionally inconsistent. Specialized tests, like neuropsychology and advanced imaging, are often restricted, especially outside academic centers.

Interpreting subtle clinical changes requires a comprehensive understanding of a patient’s baseline function and history, which is frequently fragmented across providers and systems. When clinicians suspect progression, they need confirmatory visits and additional assessments before updating the disease status.

Implementing real-time analytics, artificial intelligence (AI)-driven decision tools, or continuous digital monitoring is still aspirational, as most health systems lack the necessary infrastructure, reimbursement models, or regulatory approvals.^10^ Until these challenges are addressed, MS care logistical realities will prevent real-time progression detection, regardless of theoretical advancements.

## Misplaced certainty risks patient harm

Claiming that real-time detection of MS progression is currently possible is inaccurate and potentially harmful. It risks creating false expectations among clinicians, patients, and policymakers, potentially leading to inappropriate treatment escalations, diagnostic overreach, or missed chances for meaningful longitudinal assessment.

Real-time detection implies making immediate decisions that have significant consequences, such as switching therapies or documenting progression for insurance purposes, all of which require high diagnostic confidence. Without validated methods for instantaneous confirmation, premature action may harm.

A serial approach with data-informed recognition of the need for time and context provides a more reliable means of identifying progression. While this method may lack the immediacy of “real-time” detection, it remains the gold standard of clinical care until more effective tools are developed and validated.

## Conclusion

Earlier, more precise detection of MS progression is a key goal in modern care. However, the claim that real-time detection is feasible in current clinical environments lacks support from existing evidence or practice. Factors such as biological complexity, limitations of current outcome measures, the developmental stage of digital tools, and real-world constraints render this goal unattainable for now.

While future advancements in technology, algorithms, and integrated care hold promise, we must avoid confusing potential with reality. Currently, real-time detection of progression is more of an aspirational ideal than an achievable standard. Acknowledging this is not a failure; rather, it reflects a commitment to scientific rigor, patient safety, and ongoing improvement in MS care.

## References

[1] Inojosa H Ziemssen T. How to reduce the delay of diagnosing secondary progression in multiple sclerosis. Mult Scler 2021; 27(4): 646–647. 32697175 10.1177/1352458520943799

[bibr2-13524585251409547] Kuhlmann T Moccia M Coetzee T , et al. Multiple sclerosis progression: Time for a new mechanism-driven framework. Lancet Neurol 2023; 22(1): 78–88. 36410373 10.1016/S1474-4422(22)00289-7 PMC10463558

[3] Absinta M Lassmann H Trapp BD. Mechanisms underlying progression in multiple sclerosis. Curr Opin Neurol 2020; 33: 277–285. 32324705 10.1097/WCO.0000000000000818 PMC7337978

[4] Dillenseger A Weidemann ML Trentzsch K , et al. Digital biomarkers in multiple sclerosis. Brain Sci 2021; 11: 1519. 34827518 10.3390/brainsci11111519 PMC8615428

[5] Inojosa H Proschmann U Akgün K , et al. Should we use clinical tools to identify disease progression? Front Neurol 2021; 11: 628542. 33551982 10.3389/fneur.2020.628542 PMC7859270

[6] Giovannoni G Popescu V Wuerfel J , et al. Smouldering multiple sclerosis: The “real MS.” Ther Adv Neurol Disord 2022; 15: 17562864211066751. 10.1177/17562864211066751 PMC8793117 35096143

[7] Scalfari A Traboulsee A Oh J , et al. Smouldering-associated worsening in multiple sclerosis: An international consensus statement on definition, biology, clinical implications, and future directions. Ann Neurol 2024; 96(5): 826–845. 39051525 10.1002/ana.27034

[8] Yousef H Malagurski Tortei B Castiglione F. Predicting multiple sclerosis disease progression and outcomes with machine learning and MRI-based biomarkers: A review. J Neurol 2024; 271(10): 6543–6572. 39266777 10.1007/s00415-024-12651-3 PMC11447111

[9] Bsteh G Krajnc N Altmann P , et al. Treating to target in multiple sclerosis: Do we know how to measure whether we hit it? Eur J Neurol 2024; 31(12): e16526. 10.1111/ene.16526 PMC11554867 39445673

[10] Inojosa H Voigt I Wenk J , et al. Integrating large language models in care, research, and education in multiple sclerosis management. Mult Scler 2024; 30(11–12): 1392–1401. 39308156 10.1177/13524585241277376 PMC11514324

